# Actionable Genes and Carcinogenic Pathways for Gastric Cancer in Latinos

**DOI:** 10.1002/cam4.71216

**Published:** 2025-09-09

**Authors:** Ingrid M. Montes‐Rodríguez, Hilmaris Centeno‐Girona, Sol V. Pérez‐Mártir, Noridza Rivera, Marcia Cruz‐Correa

**Affiliations:** ^1^ Division of Clinical & Translational Cancer Research, Medical Sciences Campus University of Puerto Rico Comprehensive Cancer Center San Juan Puerto Rico; ^2^ Division of Hematology & Oncology, Department of Medicine, Medical Sciences Campus University of Puerto Rico School of Medicine San Juan Puerto Rico; ^3^ Division of Gastroenterology, Department of Medicine University of Puerto Rico School of Medicine San Juan Puerto Rico

**Keywords:** gastric cancer, GENIE, Hispanics, molecular profiling, precision oncology, TCGA

## Abstract

**Background:**

Gastric cancer (GC) is the fourth leading cause of cancer‐related death globally. Tumor profiling has revealed actionable gene alterations that guide treatment strategies and enhance survival. Among Hispanics living in Puerto Rico (PRH), GC ranks among the top 10 causes of cancer‐related death. However, the genetic mutational landscape of GC tumors from PRH remains unexplored. This study aimed to identify the most prevalent genetic alterations in GC tumors among PRH.

**Methods:**

We examined tumor mutational profiles of GC from 106 PRH between 2015 and 2022 (provided by CARIS Life Sciences and the Precision Oncology Alliance). Next‐generation sequencing data were available for 85 cases, which were categorized as hypermutated (≥ 10 mutations/megabase) or non‐hypermutated (< 10 mutations/megabase).

**Results:**

Among the non‐hypermutated cases, the most frequently mutated genes were *TP53* (56.9%), *CDH1* (29.2%), *ARID1A* (27.4%), and *KMT2D* (25.7%). Compared to TCGA, a majority non‐Hispanic cohort, PRH had significantly higher mutational frequencies in driver genes in both intestinal type (*TP53*, *CBLB*, and *MYH11*) and diffuse type (*CDH1*, *ARID1A*, and *KMT2D*) GC.

**Discussion:**

Intestinal‐type GC in PR aligns with the chromosomal instability (CIN) molecular classification, showing a higher frequency of *TP53* mutations than TCGA, potentially indicating more aggressive tumor biology and poorer prognosis. Diffuse‐type GC showed higher *CDH1* mutations, correlating with the genomically stable (GS) classification, characterized by fewer chromosomal changes but significant genetic alterations, including those in *ARID1A* and *KMT2D*.

**Conclusions:**

This study shows that the unique genetic landscape of GC tumors in this group may lead to more aggressive cases and affect treatment responses, contributing to higher mortality rates. The higher mutation rates in biomarkers related to prognosis and therapy suggest that further research might uncover additional susceptibility variants. This underscores the importance of including Hispanics in genomic studies to better understand the genetic pathways associated with the risk and progression of GC.

## 1 Introduction

Gastric cancer (GC) is the fourth leading cause of cancer‐related deaths globally [1]. However, significant differences in GC mortality exist among racial and ethnic groups, including differences among Hispanic subgroups. For instance, Hispanics living in Puerto Rico (PRH) have a higher GC mortality rate than those in the mainland United States [2, 3], especially among those under 50, who show greater annual increases in rates compared to other ethnic groups [3]. These differences may stem from a complex interplay of genetic predisposition and environmental factors [4]. Key risks include chronic infections with *Helicobacter pylori* or Epstein–Barr virus (EBV), along with lifestyle factors like high salt intake, alcohol use, and smoking [5, 6].

GC can be histopathologically classified as intestinal or diffuse [7]. The intestinal‐type GC often arises from pre‐existing lesions (e.g., intestinal metaplasia) and is linked to *H. pylori* infection. It is more common among older adults, with a higher prevalence in males, and is associated with dietary risk factors and chronic conditions such as atrophic gastritis, leading to an earlier diagnosis and generally better prognosis [8, 9, 10]. In contrast, diffuse‐type GC is associated with highly migratory and poorly differentiated cells [11]. This subtype predominantly affects younger individuals, mostly females, and is associated with germline *CDH1* mutations, contributing to more aggressive disease, late‐stage diagnosis requiring more intensive treatment, and poorer outcomes [4, 8]. In contrast, in sporadic GC, *CDH1* inactivation is frequently attributed to promoter hypermethylation [12].

In addition to histological subtypes, GC may also be classified into four distinct molecular subtypes: microsatellite instability high (MSI‐H), chromosomal instability (CIN), genomically stable (GS), and EBV‐positive [13]. A comprehensive understanding of these molecular subtypes is essential for the advancement of targeted therapies.

Despite numerous studies leveraging genetic profiling to classify GC and identify driver mutations, there remains a significant gap in knowledge regarding comprehensive genetic assessment of tumors from PRH. This study aimed to address this gap by presenting, for the first time, an extensive characterization of somatic mutations among GC cases from PRH and comparing these findings to other publicly available national cohorts.

## 2 Methodology

We evaluated the mutational profiles of 106 GC tumors from PRH who underwent genomic testing between 2015 and 2022. The data were collected and provided by the Precision Oncology Alliance (POA) [14] using the CARIS Life Sciences next‐generation sequencing (NGS) platform, which employed either a 592‐gene panel or whole‐exome sequencing (WES) to detect mutations, indels, and copy number amplification (CNA). To prevent bias, only genes covered by both sequencing methods were included in the analysis. In some samples, genes were ruled indeterminate due to the low coverage of some or all exons. Copy number deletion analysis was added to the CARIS WES platform in late 2021, rendering deletion data unavailable for many patients. To ensure consistency and avoid bias, we excluded this data from the analysis. Whole transcriptome sequencing (WTS) was performed to identify gene fusions. Additional details regarding next‐generation sequencing (NGS), CNA, WTS, tumor mutational burden (TMB), and immunohistochemical (IHC) methodologies are provided in the Supporting Information Methodology.

CARIS analyzed formalin‐fixed, paraffin‐embedded (FFPE) tissue samples. Tumor blocks were sectioned, and slides were reviewed by a board‐certified pathologist or a trained pathologist assistant. Tumor enrichment was performed through manual microdissection to selectively isolate targeted tissue for DNA/RNA sequencing. CARIS analyzes only tumor tissue; matched non‐tumor samples were not included in the analysis.

We conducted a comparative analysis of somatic mutations in GC tumors from PRH with those Stomach Adenocarcinoma cases from The Cancer Genome Atlas (TCGA) Firehose Legacy Data and from the AACR Project GENIE (public release version 9.0), both available in the cBioPortal for Cancer Genomics [15, 16]. TCGA's database had 478 cases, with only 1% identifying as Hispanic; hence, the data were not segregated by ethnicity. The GENIE database had 188 cases of Hispanics; these cases were taken as an independent subset to compare PRH with the Hispanics in GENIE (GENIE‐H) and the 1021 cases available for GENIE‐Non‐Hispanics (GENIE‐NH). We downloaded the somatic mutation and CNA data from cBioPortal, selecting only one sample per patient. Data on gene fusions were unavailable.

### 2.1 Statistical Analysis

#### 2.1.1 Overall Mutational Frequency Comparisons

We used descriptive statistics to characterize the dataset in this study. The equality of the proportions between our study population and TCGA, GENIE‐NH, and GENIE‐H cohorts was evaluated using Chi‐square tests; Fisher's exact test was used when expected counts were < 5. A *p* < 0.05 was considered statistically significant. All data analyses were performed in R (v4.4.1).

### 2.2 Data Availability

Tables with all mutational data for PRH are included as a Supporting Information. The TCGA and GENIE datasets are publicly available in cBioPortal [17].

## 3 Results

Table 1 provides an overview of the clinical characteristics of the overall PRH cohort, including classification by TMB. Of the overall cohort (*n* = 106), 84 cases had NGS data, with 20 cases sequenced using the CARIS 592‐gene panel and 64 cases sequenced using WES. Of these, two lacked TMB data. Based on the standard clinical threshold [18], the remaining 82 cases were categorized as hypermutated (high TMB, ≥ 10 mutations/megabase) or non‐hypermutated (low TMB, < 10 mutations/megabase). Stratification by TMB is essential to prevent potential bias in mutational frequency analysis, as hypermutated tumors can artificially inflate mutation frequencies and obscure biological differences between groups. To address this, we present an overview of the results for hypermutated cases (*n* = 10), followed by a more focused analysis of non‐hypermutated cases (*n* = 72) to better understand their distinct mutational profiles and clinical implications. Statistically significant differences were identified between the hypermutated and non‐hypermutated groups with respect to the Lauren classification.

**TABLE 1:** Clinical characteristics of PRH cohort.

Clinical characteristics	Overall (*n* = 106)	Non‐hypermutated (*n* = 72)	Hypermutated (*n* = 10)	*p*
	No. (%)	No. (%)	No. (%)	
Gender				
Male	55 (51.9)	38 (52.7)	7 (70.0)	0.500
Female	51 (48.1)	34 (47.2)	3 (30.0)	
Median age	65.5	66	68.5	
< 50	16 (15.1)	12 (16.7)	2 (20.0)	0.677
≥ 50	90 (84.9)	60 (83.3)	8 (80.0)	
Stage				
I	2 (1.9)	2 (2.7)	0 (0.0)	0.209
II	6 (5.7)	3 (4.2)	2 (20.0)	
III	11 (10.4)	8 (11.1)	2 (20.0)	
IV	37 (34.9)	24 (33.3)	3 (30.0)	
Unknown	50 (47.2)	35 (48.6)	3 (30.0)	
Lauren classification				
Diffuse	42 (39.6)	29 (40.3)	1 (10.0)	0.023
Intestinal	32 (30.2)	18 (25.0)	7 (70.0)	
Unknown	32 (30.2)	25 (34.7)	2 (20.0)	
Location				
Cardia + Fundus	17 (16.0)	13 (18.1)	2 (20.0)	0.255
Body	67 (63.2)	45 (62.5)	4 (40.0)	
Antrum	22 (20.8)	14 (19.4)	4 (40.0)	

Among the 106 samples analyzed, there was nearly equal representation of men and women. The median age was 65.5 years, with most patients being 50 years or older. Of the 74 PRH cases with staging data available, stage IV cancer was the most common, followed by stage III, II, and I. According to the histological Lauren classification, diffuse‐type tumors accounted for more cases than intestinal‐type tumors. However, a substantial percentage of tumor cases lacked histological classification. Most tumors were located in the body of the stomach, followed by the antrum, with a smaller proportion in the fundus/cardia. These patterns were also reflected in the TMB‐based subgroups of the cohort.

### 3.1 Mutational Landscape of GC Tumors From PRH

#### 3.1.1 Overall Mutational Profile

NGS data were available for 84 of 106 PRH GC cases. Commonly mutated genes included *TP53*, *ARID1A*, *CDH1*, *KMT2D*, *LRP1B*, *RECQL4*, and *CSF3R* (Table 2). Compared to reference datasets, PRH GC tumors exhibited higher mutational frequencies for most of the top mutated genes. CNA and gene fusions are discussed in the subsequent sections, following stratification by TMB status.

**TABLE 2:** Overall top mutated genes in gastric cancer by cohort.

Gene	PRH			TCGA				GENIE‐H				GENIE‐NH			
	*n*	Tot	MF, %	*n*	Tot	MF, %	*p*	*n*	Tot	MF, %	*p*	*n*	Tot	MF, %	*p*
*TP53*	46	84	54.8	190	478	39.7	0.011	90	184	48.9	0.374	534	947	56.4	0.831
*ARID1A*	24	73	32.9	102	478	21.3	0.023	33	155	21.3	0.059	184	921	20.0	0.009
*CDH1*	25	84	29.8	33	478	6.9	0.000	23	183	12.6	0.001	174	945	18.4	0.008
*KMT2D*	23	82	28.0	69	478	14.4	0.001	29	155	18.7	0.098	119	920	12.9	0.000
*LRP1B*	16	84	19.0	104	478	21.8	0.548	6	36	16.7	0.757	9	62	14.5	0.472
*RECQL4*	9	51	17.6	17	478	3.6	0.002	0	148	0.0	0.000	0	832	0.0	0.000
*CSF3R*	8	54	14.8	12	478	2.5	0.001	2	135	1.5	0.002	27	849	3.2	0.000
*KMT2C*	12	83	14.5	55	478	11.5	0.517	9	126	7.1	0.085	48	584	8.2	0.054
*BRCA2*	11	84	13.1	37	478	7.7	0.123	11	154	7.1	0.130	66	920	7.2	0.041
*SPEN*	10	84	11.9	37	478	7.7	0.232	13	131	9.9	0.647	28	651	4.3	0.005
*ERBB4*	10	84	11.9	49	478	10.3	0.605	7	172	4.1	0.018	63	947	6.7	0.097
*IRS2*	9	76	11.8	8	478	1.7	0.000	3	132	2.3	0.010	22	652	3.4	0.002
*RPTOR*	6	52	11.5	16	478	3.3	0.009	2	147	1.4	0.001	17	861	2.0	0.001
*MYH9*	9	83	10.8	35	478	7.3	0.208	2	21	9.5	0.999	2	39	5.1	0.500
*SMARCA4*	9	84	10.7	25	478	5.2	0.072	13	155	8.4	0.438	48	921	5.2	0.041
*CLTCL1*	7	69	10.1	12	478	2.5	0.010	0	1	0.0	0.999	0	3	0.0	0.999
*KDM5A*	8	80	10.0	23	478	4.8	0.112	8	146	5.5	0.296	29	858	3.4	0.006
*CACNA1D*	7	72	9.7	32	478	6.7	0.391	NP				NP			
*ERCC5*	8	83	9.6	9	478	1.9	0.002	3	138	2.2	0.022	16	817	2.0	0.001
*MYH11*	7	73	9.6	25	478	5.2	0.165	0	1	0.0	0.999	0	22	0.0	0.195
*APC*	8	84	9.5	49	478	10.3	0.884	9	183	4.9	0.152	74	945	7.8	0.635
*PIK3CA*	8	84	9.5	65	478	13.6	0.264	27	184	14.7	0.205	94	947	9.9	0.882
*ATR*	8	84	9.5	29	478	6.1	0.239	7	148	4.7	0.154	24	863	2.8	0.007
*ECT2L*	8	84	9.5	0	478	0.0	0.000	0	1	0.0	0.999	0	14	0.0	0.597
*GRIN2A*	8	84	9.5	21	478	4.4	0.047	4	126	3.2	0.069	23	596	3.9	0.047
*KMT2A*	8	84	9.5	38	478	7.9	0.627	7	155	4.5	0.198	37	923	4.0	0.046
*RET*	8	84	9.5	15	478	3.1	0.010	4	184	2.2	0.011	27	947	2.9	0.006
*ROS1*	8	84	9.5	24	478	5.0	0.122	5	155	3.2	0.069	29	921	3.1	0.007
*ZNF521*	8	84	9.5	31	478	6.5	0.394	0	1	0.0	0.999	0	9	0.0	0.999

Abbreviations: MF, mutational frequency; *n*, samples with mutation; Tot, total number of samples.

#### 3.1.2 Hypermutated GC Cases

All data related to the hypermutated cases can be found in Supporting Information File A. The most frequently mutated genes among the 10 patients with hypermutated GC tumors were *KMT2C* (70.0%), *LRP1B* (70.0%), and *KMT2A/KMT2D/PMS1/TP53* (50.0%), with amplifications of *ARNT*, *MCL1*, *CDX2*, *FOXO1*, and *SMARCB1* observed in 10% of the cases. Gene fusions were profiled in nine hypermutated cases, and four patients had single gene fusions, including *ZMIZ1‐POLR3A*, *NLK‐SSH2*, *SLC12A2‐CCDC192*, and *TIMM23‐BMS1P4*. One patient had > 20 gene fusions.

Only five of the 81 cases with available MSI status data (6.2%) were identified as MSI‐H, and all five exhibited hypermutation. Within the MSI‐H subgroup, mutations in *KMT2C* and *ARID1A* were observed in all cases. Furthermore, the genes *KMT2D*, *CRTC1*, *PRKDC*, and *LRP1B* had mutation frequencies of 80%.

#### 3.1.3 Non‐Hypermutated GC Cases

##### 3.1.3.1 Top Genomic Alterations Among Non‐Hypermutated GC Cases by Anatomical Location

**FIGURE 1:**
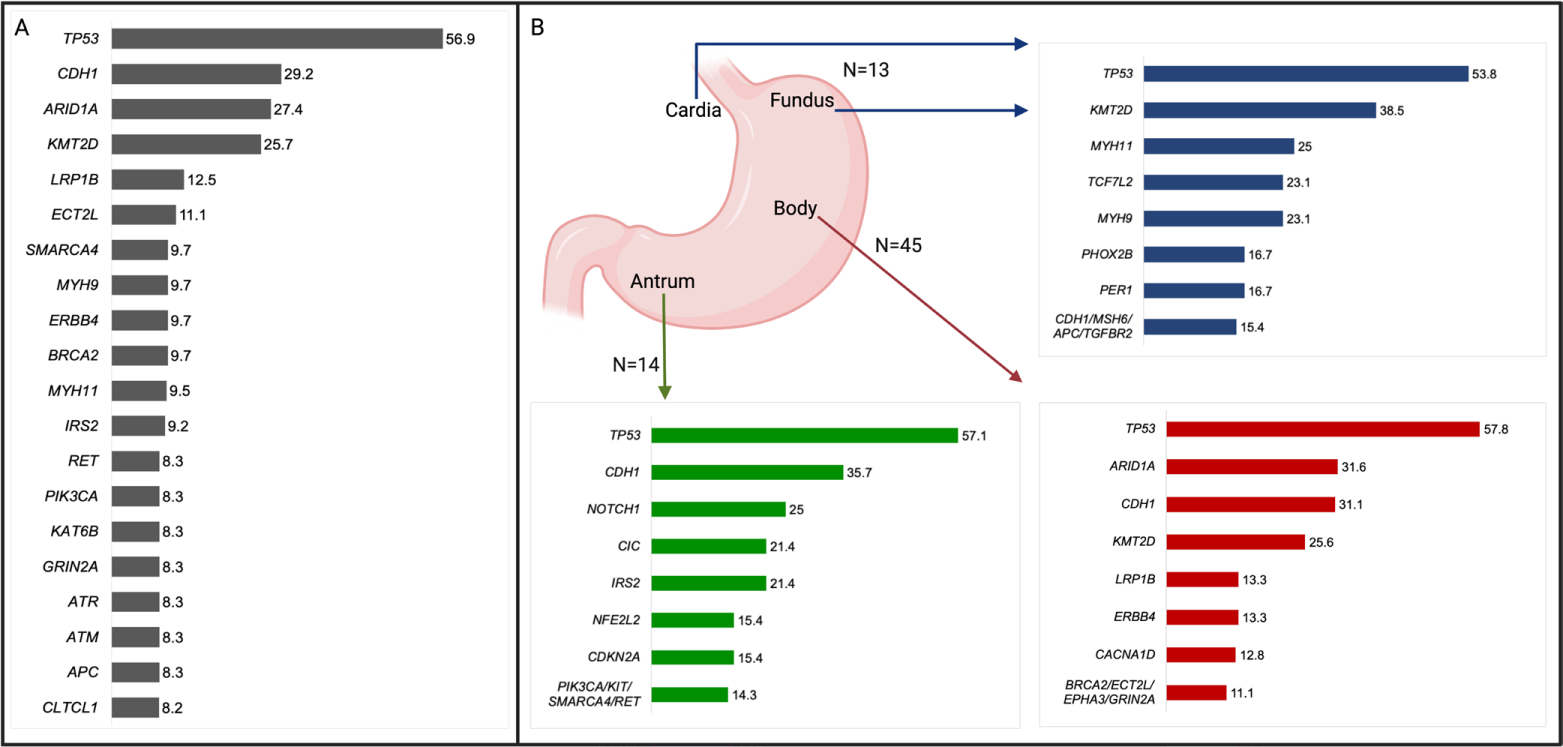
(A) Mutational frequencies of the top mutated genes identified in the 72 non‐hypermutated gastric cancer (GC) tumors from PRH (*n* = 72 for all genes except *ARID1A* (*n* = 62), *KMT2D* (*n* = 70), *MYH11* (*n* = 63), *IRS2* (*n* = 65), and *CLTCL1* (*n* = 61)). (B) Mutational frequencies by anatomical location of non‐hypermutated GC tumors from PRH. For Fundus/Cardia, there were 13 cases, and *n* = 13 for all genes, except for *MYH11*, *PHOX2B*, and *PER1*, which had *n* = 12. For the Body, there were 45 cases, and all genes had *n* = 45, except *for ARID1A* (*n* = 38), *KMT2D* (*n* = 43), and *CACNA1D* (*n* = 39). For the Antrum, there were 14 cases, and all genes had *n* = 14, except for *NOTCH1* (*n* = 12), *CDKN2A* (*n* = 13), and *NFE2L2* (*n* = 13).

All data related to the non‐hypermutated cases can be found in Supporting Information File B. The frequencies of the top mutated genes among the 72 non‐hypermutated GC tumors in the PRH cohort are depicted in Figure 1A. The most frequently mutated genes in this group were *TP53*, *CDH1*, *ARID1A*, *KMT2D*, and *LRP1B*.

Figure 1B shows the mutation frequencies by tumor location. In antral tumors, the most frequently mutated genes included *TP53*, *CDH1*, and *NOTCH1*. Body tumors exhibited high frequencies of *TP53*, *ARID1A*, and *CDH1* mutations. In the fundus/cardia, *TP53* was the most frequently mutated gene, followed by *KMT2D* and *MYH11*. *TP53* and *KMT2D* mutations were consistently high across all regions, whereas *CDH1* mutations were most frequent in the antrum and body.

*ERBB2* amplification was the most common, found in 6 of 72 cases (8.3%). Two were located in the antrum and four in the body. *KRAS* was also found in six out of 72 cases (8.3%), with four in the body and two in the fundus/cardia. *CCNE1* was detected in three out of 71 cases (4.2%), with one each in the antrum, body, and fundus/cardia. *SMARCE1* was detected in three out of 72 cases (4.2%), with two in the antrum and one in the body. Please refer to Supporting Information File B for more details.

The *CLDN18‐ARHGAP26* gene fusion was the most frequently identified among non‐hypermutated gastric tumors in the PRH cohort, occurring in 7.7% of the samples (*n* = 65), with three cases located in the body and two in the antrum.

**FIGURE 2:**
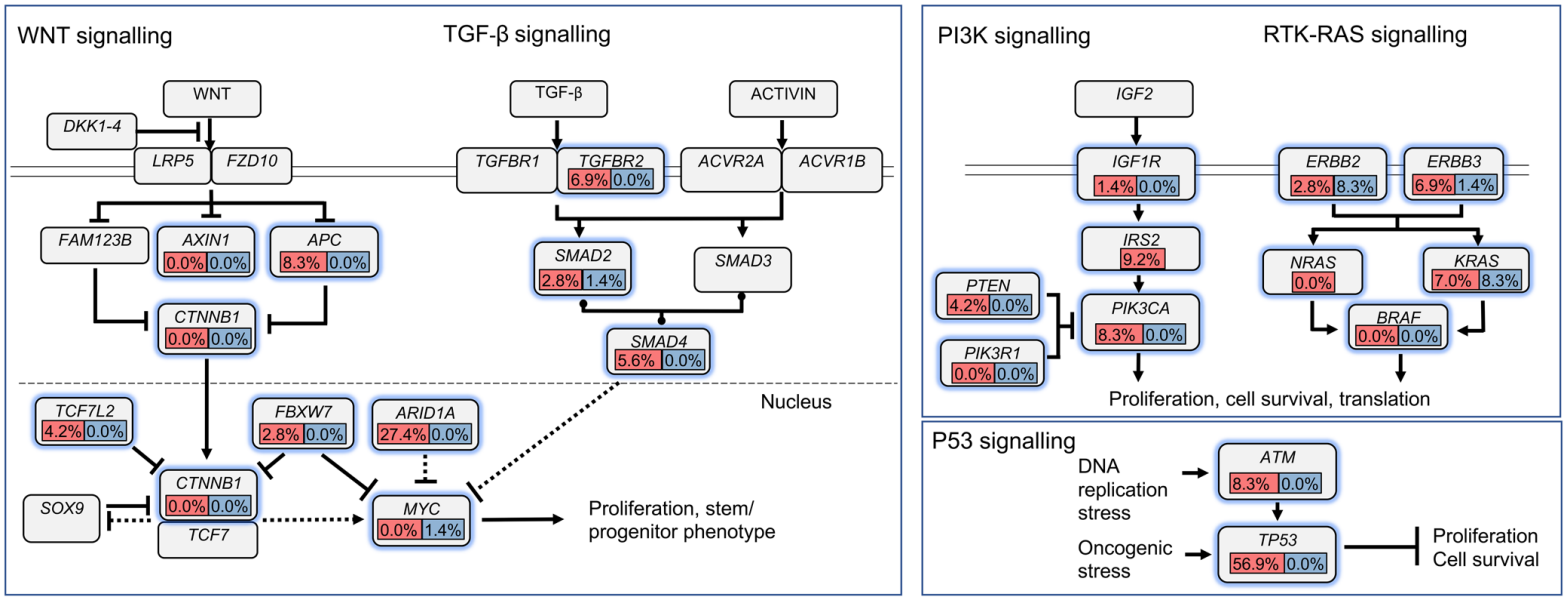
Frequency of genetic alterations in key signaling pathways in non‐hypermutated gastric cancer cases leading to deregulation of the WNT/β‐catenin, MAPK, PI3K, TGF‐β, and p53 signaling pathways in the non‐hypermutated cases. The frequencies of alterations are expressed as a percentage of all cases. The red color denotes the overall mutational frequency, and blue denotes the amplification frequency for each gene. Figure adapted from: Nature, volume 487, pages 330–337 (2012).

Figure 2 depicts the frequency of the most common genetic alterations identified in GC tumors from PRH, emphasizing their potential influence on the deregulation of key signaling pathways, including WNT/β‐catenin, MAPK, PI3K, TGF‐β, and p53 pathways.

##### 3.1.3.2 Top Genomic Alterations Among Non‐Hypermutated GC Cases by Histologic Subtype

**FIGURE 3:**
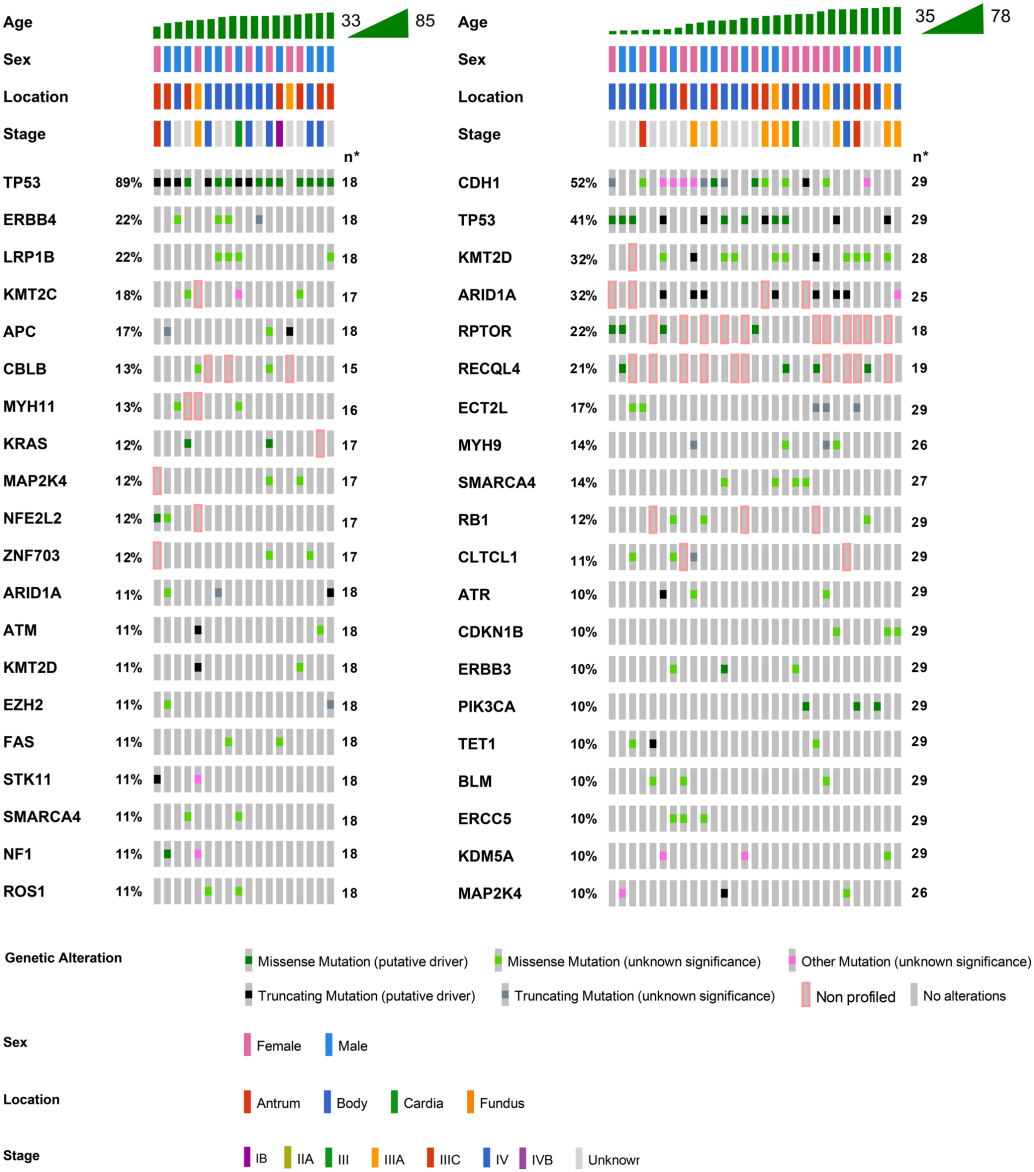
Genetic landscape of non‐hypermutated intestinal and diffuse types of GC. Landscape of significantly mutated genes in intestinal and diffuse tumors.

Figure 3 illustrates the genetic alterations and clinical characteristics of the intestinal‐ and diffuse‐type GC cases within the PRH cohort. In the intestinal‐type GC group, *TP53* was the most frequently mutated gene, followed by *ERBB4* and *LRP1B*. Other significant mutations in this group included *KMT2C*, *APC*, *CBLB*, and *MYH11*. The genetic alterations were primarily missense mutations, with a minority of truncating and in‐frame mutations. In the diffuse‐type group, *CDH1* mutations were predominant, present in 52% of cases, followed by *TP53* and *KMT2D*. Mutations in *ARID1A*, *RPTOR*, *RECQL4*, and *ECT2L* were also observed. Similar to those in intestinal‐type GC, missense mutations were the most common genetic alterations.

All mutational frequencies, CNAs, gene fusions, and subtype comparisons are detailed in Supporting Information File C. Mutations in several genes were observed in both subtypes, but only *TP53*, *CDH1*, and *KMT2C* showed significant differences: *TP53* was more common in intestinal type, *CDH1* in diffuse type, and *KMT2C* mutations were exclusive to intestinal type tumors.

The mutation types also varied between the subtypes. In both groups, *TP53* mutations were predominantly missense; however, intestinal‐type GC had a higher proportion of truncating mutations (33.3% vs. 17.2%). *ARID1A* mutations comprised a combination of truncating and missense mutations in both subtypes, with truncating mutations being more prevalent in diffuse‐type GC (28.0% vs. 11.1%). Similarly, *KMT2D* mutations were primarily missense mutations in both subtypes.

Regarding gene amplification (Supporting Information File C), intestinal‐type GC had higher amplification rates for *ERBB2* (16.7%, *n* = 18), *CCNE1* (11.8%, *n* = 17), and *KRAS* and *SMARCE1* (11.1%, *n* = 18). In diffuse‐type GC, several genes, including *FGF3*, *HMGA2* (3.6%, *n* = 28), and *BCL11A*, *CCND1*, *CCND2*, *FGF19*, *FGF4*, *FOXA1*, *KRAS*, *MDM2*, *and NFKBIA* (3.4%, *n* = 29), showed similar frequencies. No significant differences were observed between histologic subtypes regarding CNA.

In cases of intestinal‐type GC tumors, gene fusions involving *ARHGAP26*, *PKN1*, and *PRKCA* (specifically *CLDN18:ARHGAP26*, *ZNF791:PKN1*, and *MSI2:PRKCA*) were identified in 5.9% (*n* = 17) of the cases. In diffuse‐type GC tumors, fusions involving *ARHGAP26* and *MUSK* (*CLDN18:ARHGAP26* and *SNX30:MUSK*) were detected in 12.0% and 4.0% (*n* = 25) of cases, respectively. No significant differences in gene fusion frequencies were observed between the histologic subtypes.

##### 3.1.3.3 Mutational Frequency and CNA Comparison in Non‐Hypermutated GC: PRH Vesus TCGA

We compared the mutational frequencies across the intestinal and diffuse types of GC, as reported in TCGA (Table 3). We were unable to make similar comparisons with the GENIE cohort because the database does not include TMB status. The TCGA dataset included 86 hypermutated and 478 non‐hypermutated GC cases. We compared these proportions with those of the PRH cohort and found no statistical difference between the populations.

**TABLE 3:** Top gene mutations in non‐hypermutated gastric tumors in PRH by histologic subtype compared to TCGA.

	PRH				TCGA			*p*
	Gene	*n*	Total	MF, %	*n*	Total	MF, %	
Gene mutations								
Intestinal	*TP53*	16	18	88.9	75	158	47.5	0.001
	*ERBB4*	4	18	22.2	14	158	8.9	0.094
	*LRP1B*	4	18	22.2	23	158	14.6	0.494
	*KMT2C*	3	17	17.6	9	158	5.7	0.097
	*APC*	3	18	16.7	11	158	7.0	0.159
	*CBLB*	2	15	13.3	0	158	0.0	0.007
	*MYH11*	2	16	12.5	2	158	1.3	0.043
	*KRAS*	2	17	11.8	6	158	3.8	0.176
	*MAP2K4*	2	17	11.8	2	158	1.3	0.048
	*NFE2L2*	2	17	11.8	2	158	1.3	0.048
Diffuse	*CDH1*	15	29	51.7	13	59	22.0	0.005
	*TP53*	12	29	41.4	21	59	35.6	0.597
	*KMT2D*	9	28	32.1	1	59	1.7	< 0.001
	*ARID1A*	8	25	32.0	4	59	6.8	0.005
	*RPTOR*	4	18	22.2	1	59	1.7	0.010
	*RECQL4*	4	19	21.1	0	59	0.0	0.003
	*ECT2L*	5	29	17.2	0	59	0.0	0.003
	*MYH9*	4	29	13.8	2	59	3.4	0.089
	*SMARCA4*	4	29	13.8	1	59	1.7	0.039
	*RB1*	3	26	11.5	1	59	1.7	0.083
Copy number amplifications								
Intestinal	*ERBB2*	3	18	16.7	33	157	21.0	0.999
	*CCNE1*	2	16	12.5	27	157	17.2	0.999
	*KRAS*	2	18	11.1	13	157	8.3	0.655
	*SMARCE1*	2	18	11.1	21	157	13.4	0.999
Diffuse	*HMGA2*	1	28	3.6	2	59	3.4	0.999
	*BCL11A*	1	29	3.3	Not profiled			
	*KRAS*	1	29	3.3	6	59	10.2	0.418
	*MDM2*	1	29	3.3	5	59	8.5	0.659
	*CCND2*	1	29	3.3	2	59	3.4	0.999

Abbreviations: MF, mutational frequency; *n*, samples with mutation; Total, total number of samples.

In intestinal‐type GC, *TP53* mutations were significantly more frequent in the PRH cohort than in the TCGA cohort. Other genes that followed this trend included *MYH11*, *MAP2K4*, *NFE2L2*, and *ZNF703*. In diffuse‐type GC, several genes exhibited mutations at significantly higher rates among PRH than those found in TCGA, including *CDH1*, *ECT2L*, *KMT2D*, *ARID1A*, *RPTOR*, and *SMARCA4*.

High amplification frequencies were noted in TCGA for *ERBB2*, *CCNE1*, and *SMARCE1*, whereas *KRAS* amplification was lower than that in our PRH cohort. Additionally, diffuse‐type GC showed differences in gene amplification, with PRH exhibiting lower frequencies of *KRAS*, *MDM2*, and *FGF3* than TCGA. However, no statistically significant differences in CNAs were observed for any of the histologic subtypes.

#### 3.1.4 Prevalence of Pathogenic Infections in GC Tumors From PRH

In 2020, CARIS implemented whole‐exome sequencing in its genomic pipeline, analyzing 66 cases in our PRH cohort (data not shown). This method detected over 20 pathogens, with EBV being the most common (24.2%). Other pathogens included *Salmonella enterica* (12.1%), *Bartonella* (10.6%), *Fusobacterium nucleatum* (9.1%), and *Helicobacter pylori* (4.5%). Multi‐pathogen co‐infections were also detected in this cohort, including those with two (6.1%), three (7.6%), and four (1.5%) pathogens.

#### 3.1.5 Immunohistochemical Analysis in GC Tumors From PRH

In this cohort of GC patients, IHC analysis revealed varying expression profiles for several biomarkers (Table 4). HER2, the protein product of *ERBB2*, was expressed in 4.8% of cases. Mismatch repair proteins MLH1, MSH2, MSH6, and PMS2 showed high expression rates of 95.6%, 98.9%, 97.8%, and 94.4%, respectively. Regarding PD‐L1 expression, 55 patients were tested using the 22C3 pharmDx assay and 41 patients were tested using the FDA‐approved 28‐8 pharmDx assay. Among those tested, 67.3% of patients were positive with the 22C3 assay, while 34.1% were positive with the 28‐8 assay.

**TABLE 4:** Biomarker expression by immunohistochemistry in gastric cancer patients from PRH.

Protein	Overall (*n* = 98)				Hypermutated (*n* = 10)				Non‐hypermutated (*n* = 68)			
	Pos	Neg	Tot	*F*, %	Pos	Neg	Tot	*F*, %	Pos	Neg	Tot	*F*, %
Her2/Neu	4	79	83	4.8	0	9	9	0	3	53	56	5.4
MLH1	87	4	91	95.6	5	4	9	55.6	63	0	63	100
MSH2	90	1	91	98.9	10	0	10	100	62	0	62	100
MSH6	88	2	90	97.8	9	1	10	90	62	0	62	100
PMS2	85	5	90	94.4	5	5	10	50	62	0	62	100
PD‐L1 (22c3)	37	18	55	67.3	6	3	9	66.7	26	8	34	76.5
PD‐L1 FDA (28‐8)	14	27	41	34.1	1	0	1	100	11	22	33	33.3

Abbreviations: *F*, frequency; Neg, negative protein expression; Pos, positive protein expression; Tot, total number of samples.

When stratified by TMB status, only three non‐hypermutated samples showed HER2 positive expression, with no HER2 expression observed in the hypermutated group. Conversely, all mismatch repair proteins, MLH1, MSH2, MSH6, and PMS2, were expressed in the non‐hypermutated group. In the hypermutated group, these proteins were expressed at rates of 55.6%, 100%, 90%, and 50%, respectively. PD‐L1 expression was higher in the non‐hypermutated group, with positivity rates of 76.6% using the 22C3 pharmDx assay and 33.3% using the FDA‐approved 28‐8 assay. In the hypermutated group, 66.7% of cases tested positive with the 22C3 assay, whereas only one case was evaluated with the 28‐8 assay and was positive.

## 4 Discussion

GC poses a significant health burden in the United States. Distinct groups within the population experience a higher burden of disease; for example, Hispanic groups have approximately twice the incidence and mortality rates of those observed among non‐Hispanic whites [19]. GC prognosis among Hispanic groups is poor, with only approximately 25% surviving 5 years after diagnosis (compared to 36% among the overall population) [19]. Puerto Ricans account for the second largest proportion of United States Hispanics and experience high GC mortality rates [20]. Nevertheless, little is known about the factors contributing to GC risk in this group. This study aimed to characterize the molecular landscape of GC in PRH to (1) evaluate the genomic factors contributing to the increased risk of aggressive disease and (2) profile actionable genes to inform therapeutic strategies. Our results suggest that gastric tumor subtypes are distributed among the PRH population in patterns similar to those described in previous landmark studies [13], with several key differences.

This study is based exclusively on somatic mutation data, and the germline mutational status is beyond the scope of this study. Nonetheless, we observed significant differences at the somatic level, underscoring the relevance of population‐specific tumor biology. Although the primary focus of this study was on non‐hypermutated GC tumors, we identified significant variations in the overall mutational frequencies of the most commonly mutated genes in PRH GC tumors compared to the reference cohorts. These variations may be attributable to genetic, environmental, and socioeconomic factors specific to the Hispanic populations [21, 22, 23, 24]. For example, PRH exhibits higher mutational frequencies of genes such as *ARID1A*, *CDH1*, *KMT2D*, and *RECQL4*, which are involved in pathways related to chromatin remodeling, cell adhesion, and DNA repair [25, 26, 27, 28]. Such variations could contribute to differences in tumor behavior, response to therapy, and overall prognosis, highlighting the importance of understanding population‐specific molecular profiles to improve clinical management and outcomes in Hispanics with GC. Consequently, we stratified our analysis into hypermutated and non‐hypermutated groups, focusing primarily on the latter, as non‐hypermutated tumors represent the more common mutation landscape unaffected by hypermutation mechanisms.

The hypermutated group overlapped with the MSI‐H molecular classification, representing 6.2% of the PRH cohort. The lack of mismatch repair protein expression detected by IHC in the hypermutated group corresponds to the MSI‐H GC subgroup. The MSI‐H subtype is characterized by hypermethylation of mismatch repair genes, especially MLH1, leading to gene silencing and loss of protein expression, as well as mutations in mismatch repair genes such as *MLH2*, *MSH6*, *PMS1*, and *PMS2*, resulting in mismatch repair deficiency. This combined epigenetic and genetic alteration underpins the hypermutated phenotype detectable by the absence of protein expression on IHC [29]. We then analyzed non‐hypermutated GC cases for mutational frequencies, CNAs, and gene fusions across the histological subtypes and anatomical gastric regions.

We observed key mutational alterations across several pathways in non‐hypermutated GC cases (Figure 2), with potential implications for the PRH population. The WNT/β‐catenin pathway, in which mutations in *APC* play a central role, is associated with tumor initiation, maintenance, and poor prognosis [30]. Monitoring changes in *APC* mutations during treatment can inform therapeutic adjustments. For example, in colorectal cancer, these are linked to Wnt pathway activation and a poorer immunotherapy response [31]. Combining Wnt inhibitors with immune checkpoint therapies may overcome resistance, especially in tumors reliant on Wnt signaling [32]. In the TGF‐β pathway, mutations in *TGFBR2*, *ACVR1B*, and *SMAD2‐4* promote tumor progression and enhance invasiveness through epithelial‐to‐mesenchymal (EMT) transition and are linked to resistance against therapies such as BRAF/MEK inhibitors, EGFR tyrosine kinase inhibitors (TKIs), and SMAD4‐targeted treatments [33, 34]. In HER2‐positive GC, TGF‐β‐mediated EMT contributes to trastuzumab resistance [34]. The PI3K pathway, characterized by mutations in *PIK3CA* and *PTEN*, contributes to tumor development and therapy resistance [35]. Furthermore, mutations in *PIK3CA* and loss of *PTEN* indicate hyperactivation of the PI3K/AKT/mTOR pathway, suggesting that targeted inhibitors, such as alpelisib (a PI3Kα‐specific inhibitor) [36], ipatasertib (an AKT inhibitor) [37], and everolimus (an mTOR inhibitor) [38, 39] could effectively suppress tumor growth, especially when combined with other therapies. The MAPK pathway, affected by mutations and amplifications in *KRAS* and *ERBB2*, drives cell proliferation and survival [40]. Targeted therapies in GC focus on inhibiting key components of the MAPK pathway, such as HER2 with trastuzumab for HER2‐positive tumors and MEK inhibitors for *KRAS* mutations [41]. Finally, mutations in *TP53* and *ATM* disrupt the p53 pathway, impairing cell cycle regulation and DNA repair, leading to genomic instability and poor outcomes [42]. *TP53* mutations are involved in various cancers, and restoring p53 function or targeting its pathway represents a promising therapeutic strategy. Small molecules, such as PRIMA‐1 and its derivatives, have demonstrated the potential to reactivate mutant p53, induce apoptosis, and tumor suppression [43]. However, resistance to these targeted therapies remains a significant challenge; mutations in p53 can cause conformational changes that hinder drug binding or reactivation, and alterations in p53‐regulating proteins, such as MDM2, can further impair treatment efficacy [42]. Understanding pathway‐specific mutations in this population can inform personalized therapies that improve response rates and overcome resistance to therapy.

Our findings indicate that intestinal‐type GC in PRH aligns with the CIN molecular classification, showing a higher frequency of *TP53* mutations (88.9% vs. 47.5% in TCGA). This may be linked to more aggressive tumor biology, higher stages at diagnosis, and poorer prognosis in this population [3, 44]. This intestinal‐type profile was accompanied by amplifications in *ERBB2*, *CCNE1*, and *KRAS*, supporting the notion that receptor tyrosine kinase–Ras signaling is active in this subtype [13]. Several intestinal‐type genes have a significantly higher mutation frequency in PRH tumors than in TCGA, including *CBLB*, *MYH11*, *MAP2K4*, and *NFE2L2*. *CBLB* mutations were identified in 13.3% of PRH cases but were absent in TCGA. CBLB plays a crucial role in GC by maintaining epithelial phenotype, inhibiting migration, and preventing EMT and metastasis [45]. In GC, *CBLB* mutations are linked to shorter survival times and poor responses to treatments [46]; hence, mutations in this gene have been proposed as biomarkers for GC prognosis and monitoring [46]. Mutations in the *MYH11* gene occur approximately 10 times more frequently in PRH than in TCGA. This gene produces smooth muscle myosin heavy chain protein for muscle contraction and influences cancer development through cell movement, adhesion, transport, and growth [47]. Lower *MYH11* expression in GC is indicative of poor prognosis [48]. Loss‐of‐function mutations in *MAP2K4*, a tumor suppressor and kinase involved in the MAPK signaling pathway, confer sensitivity to MEK inhibition, suggesting that GCs with these mutations might respond better to MEK inhibitor therapy, with *MAP2K4* serving as a predictive biomarker and therapeutic target [49]. *NFE2L2* encodes NRF2, a transcription factor that upregulates antioxidant and detoxification genes, promoting cancer cell survival under oxidative stress [50]. Activating mutations result in constitutive NRF2 activation, which has been associated with chemoresistance, radioresistance, and poorer clinical outcomes [51]. In GC, the expression of *NFE2L2* has been linked to several aggressive cancer characteristics, such as larger tumor size, increased tumor depth, lymph node metastasis, lymphovascular invasion, higher tumor stage, and poorer survival, and is also linked to resistance to 5‐FU–based chemotherapy [52]. These gene mutations, particularly those associated with aggressive tumor behavior and therapy resistance in the PRH population, highlight the urgent need to explore targeted therapeutic strategies that could improve clinical outcomes and personalize treatment approaches for intestinal‐type GC in PRH.

Diffuse‐type GC among PRH exhibited a higher frequency of *CDH1* mutations than that observed in TCGA (51.7% vs. 22.0%). These mutations are linked to the GS molecular classification, a subtype known for having fewer chromosomal changes but significant epigenetic and genetic alterations, including mutations in the *ARID1A* chromatin remodeling gene and the *KMT2D* histone methyltransferase gene, both of which are highly mutated in PRH diffuse‐type GC cases [53, 54]. Additionally, *CLDN18‐ARHGAP26* fusions, also characteristic of the GS classification, were predominantly found in diffuse‐type GC. It is not surprising that mutations in the *CDH1*, *ARID1A*, and *KMT2D* genes were mostly found in the body and antrum of the stomach, as most diffuse‐type cases reported in this study were in these regions.

Several genes in diffuse‐type GC were detected more frequently in PRH than in TCGA, including *CDH1*, *KMT2D*, *ARID1A*, *RPTOR*, *RECQL4*, *ECT2L*, and *SMARCA4. CDH1* mutations were more than twice as frequent in PRH than in TCGA data. These mutations lead to the loss of E‐cadherin, a critical mediator of epithelial identity and integrity. As *CDH1* mutations are also associated with lower PD‐L1 positivity in GC, these results suggest that PRH patients with diffuse‐type GC may not benefit from this type of immunotherapy [55]. On the other hand, since *CDH1* mutations are associated with alterations in other genes related to DNA damage repair and cell cycle checkpoints—*ARID1A*, *WRN*, *POT1*, *CDK12*, and *FANCC*—patients with this GC subtype may respond better to therapies that target DNA damage repair pathways, including PARP and Wee1 inhibitors [55]. Conversely, *ARID1A* mutations are associated with improved overall survival when treated with chemotherapy and PD‐1 blockade [56]. Loss of *ARID1A* expression, primarily due to frameshift mutations, is correlated with poorer outcomes, particularly in mismatch repair‐proficient, EBV‐negative cases and proximally located, larger gastric tumors [57]. Like *ARID1A*, *KMT2D* mutations carry predictive value for response to PD‐1‐based immunotherapy, and *KMT2D*‐deficient tumors in both mice and humans are characterized by increased immune infiltration [58]. Emerging evidence suggests that tumors with *KMT2D*‐inactivating mutations may be targeted with combination treatment involving glycolysis inhibitors plus DNA crosslinking agents or PARP inhibitors [27, 59]. *ECT2L* mutations have been infrequently reported in cancer [60, 61, 62] and, to our knowledge, this is the first study reporting high mutational frequency in diffuse‐type GC, suggesting a potential role in its progression. *RPTOR*, part of the mTORC1 pathway that is active in almost 60% of GC, is linked to aggressive disease and worse outcomes [63, 64]. The use of mTOR inhibitors is not typically first‐line treatments for GC, but in combination with checkpoint inhibitors might be useful for a subset of diffuse‐type GC [64]. *RECQL4* mutations in GC are linked to cisplatin resistance and poor prognosis, underscoring the complex genetic landscape impacting GC treatment and survival [65, 66]. *SMARCA4* is a crucial component of the SWI/SNF chromatin‐remodeling complex, and its mutations have been implicated in several cancers. In GC, missense mutations in *SMARCA4* can impair its tumor‐suppressive function, potentially leading to more aggressive GC. Clinically, these mutations are associated with poorer prognosis and adverse pathological features influencing tumor progression and response to targeted therapies [67, 68].

EBV‐positive GC is a unique molecular subtype that accounts for approximately 10% of GC cases globally [13]. In our study, NGS revealed EBV presence in 24.2% of PRH GC cases. It should be noted that the gold‐standard method for detecting EBV in tissue, Epstein–Barr encoding region in situ hybridization (EBER) [69], identified only two cases out of 81 (2.5%) in this cohort (data not shown). Future studies should use EBER in situ hybridization in larger cohorts to validate EBV prevalence and explore the molecular and clinical characteristics of EBV‐positive GC in this population. The link between autoimmunity and EBV‐associated GC is of particular relevance among PRH, who also have a high prevalence of autoimmune conditions [70, 71, 72]. Autoimmunity can cause persistent inflammation and gastric mucosa atrophy [73, 74], facilitating EBV infection of epithelial cells through the infiltration of infected lymphocytes, promoting oncogenesis [75].

Owing to the limitations of our molecular dataset, we were only able to approximate the classification of GC molecular subtypes, primarily establishing associations with CIN and GS. Additionally, we found that 2.5% of the cases were EBV‐positive and 6.2% exhibited high MSI‐H status, as assessed by NGS. Despite these constraints, we believe that these findings provide valuable insights into the molecular landscape of GC in PRH, highlighting important avenues for future comprehensive studies. Importantly, these molecular features may underlie differences in tumor behavior and clinical outcomes in this population, with significant variations in key genes affecting treatment response across histologic subtypes, highlighting the need for tailored research and therapeutic strategies.

In our study, NGS also revealed the presence of other microbes in gastric tumors from PRH. While Salmonella is not typically associated with GC [76], *Salmonella enterica* was detected in 12.1% of cases. The presence of *Bartonella* and *Fusobacterium nucleatum* , the latter of which is linked to colorectal cancer progression [77], in the tumor samples further adds to the growing body of evidence implicating bacterial species in the tumor microenvironment. *Helicobacter pylori* was detected in only 4.5% of cases, which is lower than expected given its well‐established role as a major risk factor for GC [78]. This could be due to limitations in detecting *H. pylori* through whole‐exome sequencing, especially among patients treated with eradication therapies [79]. The observation of co‐infections with multiple pathogens in several cases highlights the complexity of the microbial environment in GC tumors. These polymicrobial infections could have synergistic effects on carcinogenesis or tumor progression, potentially influencing disease outcomes and treatment response [80].

This study highlights the importance of mutational profiling alongside existing risk stratification tools for treatment selection in GC. For HER2‐positive GC, targeted therapies like trastuzumab are currently in use, with newer agents such as T‐DXd (trastuzumab deruxtecan) and disitamab vedotin showing promising results [81, 82]. For non‐hypermutated cases, only 5.4% exhibited positive HER2 immunohistochemistry results, indicating that only a small subset of patients may be suitable for HER2‐targeted treatment. Interestingly, the PRH cohort showed a higher prevalence of *ERBB2* CNA (8.3%) than that of HER2 protein expression detected by IHC. This suggests that more PRH patients could potentially benefit from HER2‐targeted therapies, especially considering that *ERBB2* amplification identified through NGS of tissue DNA or circulating cell‐free DNA is increasingly recognized as a reliable biomarker for predicting the efficacy of HER2‐targeted therapies in GC [83, 84] and other cancers, where it has been shown to correlate with better responses to trastuzumab, further emphasizing the clinical relevance of CNA assessment in this population [73]. A substantial proportion of PRH GC patients exhibited high PD‐L1 expression rates, 76.5% with the 22C3 assay and 33.3% using the 28‐8 assay, even among those who were non‐hypermutated and MMR proficient. While patients with high tumor mutational burden (TMB‐H), including those with MSI‐H (6.2%) and representing 12.2% of the cohort, can benefit from PD‐1/PD‐L1 checkpoint inhibitors such as nivolumab and pembrolizumab [81, 82, 85], the high PD‐L1 positivity observed in non‐hypermutated, MMR‐proficient tumors suggests that additional PRH patients beyond those with high TMB or MSI‐H may also respond favorably to these immunotherapies [86].

In 2024, the FDA approved zolbetuximab‐clzb as a first‐line treatment for adults with locally advanced unresectable or metastatic HER2‐negative gastric or gastroesophageal junction adenocarcinoma positive for CLDN18.2, in combination with chemotherapy [87]; however, a limitation of this study was the lack of immunohistochemistry data on CLDN18.2 to inform treatment decisions. Furthermore, the relatively small sample size for hypermutated cases and the limited availability of detailed immunohistochemical data on the CLDN18.2 biomarker highlight the necessity for larger, prospective studies in Hispanic populations. Such studies should incorporate comprehensive immunohistochemistry profiling to better inform personalized treatment strategies and improve the generalizability of findings across diverse populations. Future research should explore the integration of mutational, protein expression, and clinical data to develop more precise and population‐specific therapeutic approaches for Hispanic patients.

The complexity of GC genetics emphasizes the need for tailored therapies for populations with a high burden of disease like Hispanics. This study shows that the unique genetic landscape of GC tumors in this group may lead to more aggressive cases and affect treatment responses, contributing to higher mortality rates. We found higher mutational frequencies in biomarkers for prognosis and treatment response, suggesting that further investigation could reveal additional susceptibility variants. Inclusion of Hispanics in genomic studies is essential for understanding the genetic pathways linked to GC risk and progression.

## Author Contributions

**Ingrid M. Montes‐Rodríguez:** conceptualization (lead), data curation (lead), formal analysis (lead), investigation (lead), methodology (lead), writing – original draft (lead), writing – review and editing (lead). **Hilmaris Centeno‐Girona:** formal analysis (equal), methodology (equal). **Sol V. Pérez‐Mártir:** writing – original draft (supporting). **Noridza Rivera:** resources (equal). **Marcia Cruz‐Correa:** conceptualization (equal), investigation (equal), supervision (lead), writing – review and editing (lead).

## Ethics Statement

This study was conducted in accordance with the guidelines of the Declaration of Helsinki, the Belmont Report, and the U.S. Common Rule. In keeping with 45 CFR 46.101(b)(4), this study was performed utilizing retrospective, de‐identified clinical data. Therefore, this study was considered IRB exempt, and no patient consent was necessary (IRB number: 2290034250). This study was performed in strict accordance with the recommendations of data access guidelines of TCGA and AACR project GENIE datasets.

## Conflicts of Interest

M.C.‐C. receives research grants from Janssen, Genentech, Abbvie, Merck, Mirati, Pfizer, Astra Zeneca, Incyte, Astellas, Regeneron, BMS, Gilead, Astra Zeneca, Exact Sciences, and Natera. I.M.M.‐R. declares no potential conflicts of interest. H.C.‐G. declares no potential conflicts of interest. S.V.P.‐M. declares no potential conflicts of interest. N.R. declares research contracts with Janssen, Genentech, Abbvie, Merck, Mirati, Pfizer, Astra Zeneca, Incyte, Astellas, Regeneron, BMS, Gilead, Astra Zeneca; speaker for Merck and BMS.

## Supporting information


**Appendix S1:** cam471216‐sup‐0001‐AppendixS1.zip.

## Data Availability

The data supporting the findings of this study are available in the Supporting Information tables, which contain comprehensive mutational data for gastric cancer tumors from Puerto Rican Hispanics (PRH). These tables detail mutation frequencies, gene fusions, copy number amplifications, and categorize cases by anatomical location and histopathological gastric cancer subtype, as well as distinguishing between hypermutated and non‐hypermutated cases.
