# Supplementary material for: Actionable Genes and Carcinogenic Pathways for Gastric Cancer in Latinos
Source: Cancer Med. 2025 Sep 9;14(17):e71216. doi: 10.1002/cam4.71216 (PMC12420367; doi:10.1002/cam4.71216)
Supplement: Supplementary file 1 — Appendix S1: cam471216‐sup‐0001‐AppendixS1.zip. [file CAM4-14-e71216-s001.zip › cam471216-sup-0004-Supinfo1@Supplementary Methodology.docx]

**CARIS REVISED METHODOLOGY**

**Specimen Processing**

Molecular profiling was performed at Caris Life Sciences (Phoenix, AZ, USA), a College of American Pathologists (CAP)/Clinical Laboratory Improvement Amendments (CLIA)-certified laboratory. Hematoxylin and eosin (H&E) stained formalin-fixed, paraffin-embedded (FFPE) slides of the patient’s tumor underwent review by a board-certified pathologist or trained pathologist assistant. Tumor enrichment was achieved by harvesting targeted tissue using manual microdissection techniques.

- For whole exome sequencing, a minimum of 20% tumor nuclei in the area for microdissection was required, with a minimum of 20 mm^2^ dissection area.
- For whole transcriptome sequencing, a minimum of 10% tumor nuclei in the area of microdissection was required, with a minimum 10 mm^2^ dissection area.

**Next-generation sequencing methods (592 whole-gene panel and WES)**

Because this study utilized samples spanning several years, the methods (including materials and software) used for DNA and RNA profiling evolved over time. Initially, DNA was profiled using a 592 whole-gene panel prior to adoption of whole exome sequencing (WES) with targeted enrichment of 720 clinically relevant genes in 2020; whole transcriptome sequencing (WTS) was performed separately. Nucleic acid was extracted using appropriate FFPE kits for RNA, DNA, or total nucleic acid. DNA sequencing was performed using a 592-whole gene panel on the NextSeq platform or by whole exome sequencing with enrichment of 720 clinically relevant genes on the NovaSeq 6000 platform (Illumina, Inc., San Diego, CA). WTS was performed using the Illumina Novaseq 6000 platform to an average of 60M reads. Raw WTS data was demultiplexed by Illumina Dragen BioIT accelerator, trimmed, counted, PCR-duplicates removed, and aligned to human reference genome (hg19/hg38) by STAR aligner. For transcription counting, transcripts per million (TPM) molecules were generated using the Salmon expression pipeline [1]. Variants detected were mapped to reference genome (hg19/38) using the Burrows-Wheeler Aligner (BWA 0.7.17) embedded in the analysis tools licensed from Sentieon®. Bioinformatic tools including Samtools, Pindel, and snpEff were incorporated to perform variant calling functions and annotations were standardized to the Human Genome Variation Society format. Germline variants were filtered with various germline databases, such as 1000 Genomes and dbSNP. All variants were detected with >99% confidence, with a reporting threshold of 5% variant allele frequency. Genetic variants identified were interpreted by board-certified molecular geneticists and categorized as ‘pathogenic,’ ‘likely pathogenic,’ ‘variant of unknown significance,’ ‘likely benign,’ or ‘benign,’ according to the American College of Medical Genetics and Genomics (ACMG) standards. Pathogenic and likely-pathogenic variants were counted as “reportable”.

**Copy number variations (CNV) (592-gene panel and WES)**

The copy number alteration (CNA) of each exon is determined by normalizing the sequencing depth of each exon divided by the average sequencing depth of the sample, and comparing it to the pre-calibrated mean of normalized values in the training data (The mean values are re-calibrated every 60 days with up to 10,000 samples). If all exons (excluding those with known insufficient coverage) within the gene of interest have an average of ≥3 copies and the average copy number of the entire gene is ≥6 copies, the gene result is reported as amplified. If an average of ≥ 4, but < 6 copies of a gene are detected, or if the average copy number of the gene is ≥6 copies, but contains exons with an average of < 3 copies, the gene result is reported as intermediate. If an average of < 4 copies of a gene are detected, the gene result is no amplification detected.

**Tumor mutational burden (TMB) (Method for 592-gene panel and WES)**

TMB was measured by counting mutations found per tumor in the coding regions of genes analyzed (1.4 Mb for 592, 1.5 Mb for WES). For 592-gene panel, missense mutations were counted, and for WES/Hybrid, missense, nonsense, in-frame INDEL, and frameshift variants were counted. Filtering was performed to remove low quality and low depth variants or variants determined to be unreliable or unassociated with TMB. Presumed germline variants found in databases such as dbSNP151, Genome Aggregation Database (gnomAD) (RRID:SCR_014964) and found in at least 10% of training samples were also filtered. A cutoff point of ≥10 mutations per megabase (Mb) was used based on the KEYNOTE-158 pembrolizumab trial [3], which showed that patients with a TMB of ≥10 mt/Mb across several tumor types had higher response rates than patients with a TMB of <10 mt/Mb. Samples with low depth of coverage (<400x for 592 or WES) were considered indeterminate. Caris Life Sciences is a participant in the Friends of Cancer Research TMB Harmonization Project [4].

**Mismatch Repair (MMR)/Microsatellite Instability (MSI) Status**

Multiple test platforms may be used to determine the MSI or MMR status of profiled tumors, including fragment analysis (FA, Promega, Madison, WI), immunohistochemistry (IHC), and next-generation sequencing (NGS). The three platforms generate highly concordant results as previously reported [5], and in the rare cases of discordant results, the MSI or MMR status of the tumor is determined in the order of IHC, FA, and NGS.

By IHC, the tumor was considered mismatch repair deficient (MMRd) if complete absence of protein expression of any of the four proteins was observed: MLH1 (M1 antibody), MSH2 (G2191129 antibody), MSH6 (44 antibody), PMS2 (EPR3947 antibody) (Ventana Medical Systems, Inc., Oro Valley, AZ, USA). IHC was performed on a Ventana Benchmark automated slide preparation system (Ventana Medical Systems, Inc.).

Combined NGS: MSI was determined from NGS data by analyzing INDEL mutations in an optimized number of microsatellite loci depending on the NGS assay utilized (592-gene panel, WES). Loci number analyzed was >2,000 for all NGS assays. The threshold to determine MSI-high also varied depending on the NGS assay used (592: ≥46, which was adjusted to ≥21 when the assay was optimized with fewer loci examined; WES: ≥116; Hybrid: ≥39). Indeterminate results were reported for samples with low average depth of coverage (<500x for 592 and WES; <100x for Hybrid).

**Immunohistochemistry (IHC)**

IHC was performed on FFPE sections on glass slides using automated staining techniques (Dako Link 48 Autostainer or Ventana BenchMark Autostainers). Staining was scored for intensity (0 = no staining; 1+ = weak staining; 2+ = moderate staining; 3+ = strong staining) and staining percentage (0-100%). Results were categorized as positive or negative by defined thresholds specific to each marker based on published clinical literature that associates biomarker status with patient responses to therapeutic agents. A board-certified pathologist evaluated all IHC results independently.

Antibody and IHC threshold information

| **Protein** | **Positive Threshold (intensity and % cells)** |
| --- | --- |
| PD-L1 (SP142, LDT)  RRID:AB_3076192 | Intensity ≥2+ and ≥5% (tumor staining) |
| PD-L1 (22c3 or 28-8) | Gastric, gastroesophageal junction, and esophageal adenocarcinoma: Combined Positive Score (CPS) for PD-L1 was calculated as the number of PD-L1 staining cells (tumor cells, lymphocytes, macrophages) divided by the total number of viable tumor cells, multiplied by 100. 22c3: CPS≥1; 28-8: CPS≥5. 22c3 is preferred CDx stain. |
| Ventana Pathway anti-HER2/neu (4B5) (Ventana Medical Systems, Inc., Tucson, AZ, USA) | Esophageal Adeno /GEJ/Gastric (Biopsy): =3+ in ≥5 tumor cells; (Resection): =3+ and ≥10%   The Ventana Pathway anti-HER2-/neu antibody is indicated for identifying breast cancer patients who are eligible for treatment with Herceptin® (IHC 3+ or IHC 2+/ISH amplified), KADCYLA® (IHC 3+ or IHC 2+/ISH amplified) or ENHERTU® (IHC 1+ or IHC 2+/ISH non-amplified). The PATHWAY anti-HER-2/neu (4B5) is an FDA-cleared IVD device. |
| VENTANA anti-MLH1 (M1) | Intensity ≥1+ and ≥1% of cells stained |
| VENTANA anti-MSH2 (G219-1129) | Intensity ≥1+ and ≥1% of cells stained |
| VENTANA anti-MSH6 (SP93) | Intensity ≥1+ and ≥1% of cells stained |
| VENTANA anti-PMS2 (A16-4) | Intensity ≥1+ and ≥1% of cells stained |

References:

[1] Patro, R., et al., *Salmon provides fast and bias-aware quantification of transcript expression.* Nat Methods, 2017. **14**(4): p. 417-419.

[2] Talevich, E., et al., *CNVkit: Genome-Wide Copy Number Detection and Visualization from Targeted DNA Sequencing.* PLOS Computational Biology, 2016. **12**(4): p. e1004873.

[3] Marabelle, A., et al., *Association of tumor mutational burden with outcomes in patients with select advanced solid tumors treated with pembrolizumab in KEYNOTE-158.* Annals of Oncology, 2019. **30**: p. v475-532.

[4] Merino, D.M., et al., *Establishing guidelines to harmonize tumor mutational burden (TMB): in silico assessment of variation in TMB quantification across diagnostic platforms: phase I of the Friends of Cancer Research TMB Harmonization Project.* J Immunother Cancer, 2020. **8**(1).

[5] Vanderwalde, A., et al., *Microsatellite instability status determined by next-generation sequencing and compared with PD-L1 and tumor mutational burden in 11,348 patients.* Cancer Med, 2018. **7**(3): p. 746-756.
